# Poisoning among Children Visiting the Paediatric Emergency Department in a Tertiary Care Centre

**DOI:** 10.31729/jnma.8291

**Published:** 2023-10-31

**Authors:** Askal Devkota, Santosh Adhikari, Raj Kumar B.K

**Affiliations:** 1 Department of Paediatrics, Nepal Police Hospital, Panipokhari, Kathmandu, Nepal; 2 Department of Paediatrics, Kanti Children's Hospital, Maharajgunj, Kathmandu, Nepal

**Keywords:** *children*, *Nepal*, *poisoning*

## Abstract

**Introduction:**

Poisoning occurs when substances are ingested, inhaled, or absorbed through skin contact in quantities that are harmful to the body. Knowledge of the pattern and prevalence of paediatric poisoning will help us quantify the burden of poisoning. The aim of the study was to find out the prevalence of poisoning among children visiting the Paediatric Emergency Department in a tertiary care centre.

**Methods:**

A descriptive cross-sectional study was conducted among children visiting the Paediatric Emergency Department in a tertiary care centre. Data from 1 January 2020 to 31 December 2021 was collected between 15 April 2022 to 25 April 2022 from medical records. Ethical approval was taken from the Institutional Review Committee. Children aged 0 to 14 years old were included in the study. A convenience sampling method was used. The point estimate was calculated at a 95% Confidence Interval.

**Results:**

Among 12,488 children, 162 (1.30%) (1.10-1.50, 95% Confidence Interval) had poisoning. Pesticides and insecticides were the most common agents involved in poisoning 39 (24.07%).

**Conclusions:**

The prevalence of poisoning was found to be lower than other studies done in similar settings.

## INTRODUCTION

Poison refers to any agent that can kill, injure or impair normal physiological function in humans by its chemical activity.^1^ Acute poisoning in the paediatric age group is an important medical emergency. Exogenous poisoning is one of the most common reasons for children's accidents, accounting for approximately 7% of accidents involving children under the age of five.^2^ The World Health Organization (WHO) estimates that 45,000 children and young people under the age of 20 years die each year from acute poisoning.^3^

Poisonings are preventable but in order to develop any preventive measures it is crucial to understand the prevalence, pattern and risk factors.^4^ Knowledge of these variables would allow us to understand the current situation in our area and to identify specific preventive strategies.^5^

The aim of the study was to find out the prevalence of poisoning among children visiting the Paediatric Emergency Department in a tertiary care centre.

## METHODS

A descriptive cross-sectional study was conducted among children visiting the Paediatric Emergency Department of Kanti Children's Hospital, Maharajgunj, Kathmandu, Nepal after obtaining ethical approval from the Institutional Review Committee (Reference number: 908). Data from 1 January 2020 to 31 December 2021 was collected between 15 April 2022 to 25 April 2022 from medical records. Children of age group 0 to 14 years of age with complete data were included in the study. The patients with incomplete medical records were excluded from the study. A convenience sampling method was used. Sample size was calculated by using the following formula:


$$\begin{array}{ll} \text{n} & ={\text{Z}}^{2}\times \frac{\text{p}\times \text{q}}{{\text{e}}^{\text{2}}}\\ & =1.96^{2}\times \frac{0.50\times 0.50}{0.01^{2}}\\ & =9604 \end{array}$$

Where,

n = minimum required sample size
Z = 1.96 at 95% Confidence Interval (CI)
p = prevalence taken as 50% for maximum sample size
q = 1-p
e = margin of error, 1%

The calculated sample size was 9,604. However, 12,488 samples were taken for the study.

The age, sex, timing of poisoning, type of poisoning, and need for ICU admission were collected from medical records. Data collection was done by reviewing the history of physical examination of patients. A standardized questionnaire was used as proforma to collect all the relevant information. The type of substance involved and timing were carefully filled in the questionnaire form after which poisoning was diagnosed.^5^

Data analysis was done using IBM SPSS Statistics version 23.0. The point estimate was calculated at a 95% CI.

## RESULTS

Among 12,488 children, 162 (1.30%) (1.10-1.50, 95% CI) had poisoning. Among them 98 (60.50%) were males and 64 (39.50%) were females with male to female ratio of 1.53. The mean age of presentation was 4.86±4.11 years. The highest rate of poisoning was in children of age group 1-4 years, 94 (58.02%) (Table 1).

**Table 1:** Age-wise distribution children with poisoning (n= 162).

Age group (years)	n (%)
<1	8 (4.94)
1-4	94 (58.02)
5-12	43 (26.55)
13-14	17 (10.49)

Most poisonings occurred during the afternoon, 56 (34.57%) (Table 2).

**Table 2:** Time of presentation of poisoning (n= 162).

Time	n (%)
Morning (5 am-12 pm)	23 (14.19)
Afternoon (12-5 pm)	56 (34.57)
Evening (5-9 pm)	41 (25.31)
Night (9 pm-4 am)	42 (25.93)

Pesticides and insecticides werethe most commonagent involved in poisoning 39 (24.07%) (Table 3).

**Table 3:** Types of poison (n = 162).

Types of poison	n (%)
Pesticides and insecticides	39 (24.07)
Envenomation	33 (20.37)
Medications	19 (11.73)
Hydrocarbons	14 (8.65)
Plants	8 (4.94)
Unknown	24 (14.8)
Others	25 (15.43)

The most frequent single agent leading to presentation and intensive care unit (ICU) admission was organophosphate 39 (24.07%). A total of 80 (49.39%) were discharged after a few hours of observation and around one-third of patients, 60 (37.03 %) required ICU admission (Table 4).

**Table 4:** Outcomes of the children with poisoning (n = 162).

Outcome	n (%)
Discharge	80 (49.39)
ICU	60 (37.03)
Leave against medical advice (LAMA)	22 (13.58)

## DISCUSSION

In our study, among 12,488 children, 1.30% had poisoning which is higher as compared to similar studies done abroad, 0.38%^3^ but lower than the similar study done in Nepal 4.3%.^6^

There were 94 (58.02%) children between 1-5 years of age. In a similar study done in Iran, 58 (55.20%) children were of age group 1-5 years probably because this is the age group of exploration.^7^ In the current study, most of the children were male which was similar to the studies in Nigeria 69 (61.06%) and Taiwan province 301 (52.30%).^8,9^ Males are probably more active and curious than females, and in most societies are less under parental supervision, which may justify the high rate of accidents, including poisoning in males.

In the present study pesticides and insecticides were the most common cause of poisoning. Similar findings were reported in a previous study done in Nepal 73 (59.90%).^6^ However kerosene was the most common cause of poisoning in similar studies done in Delhi 11 (21.10%) and South India 95 (28.61%).^10,11^ A similar study done in Australia revealed paracetamol was the most common cause of poisoning, 159 (10.48%).^2^

The main limitation of the study is that it is single centre study, hence the result could not be generalized to a larger setting.

## CONCLUSIONS

The prevalence of poisoning was lower than other studies done in similar settings. It would allow us to understand the current situation in our area and identify specific preventive strategies.
